# Weaning from mechanical ventilation in ICU patients: research hotspots and trends in the past decade—a bibliometric analysis

**DOI:** 10.3389/fmed.2026.1790796

**Published:** 2026-04-10

**Authors:** Yuanyuan Mi, Meng Liu, Hao Li, Xuyang Ye, Jiang Dong, Jing Liu, Xing Zhao

**Affiliations:** 1 Department of Intensive Care Medicine, The Sixth Hospital of Wuhan, Affiliated Hospital of Jianghan University, Wuhan, China; 2 Evidence-based Nursing Research Center, The Sixth Hospital of Wuhan, Affiliated Hospital of Jianghan University, Wuhan, China; 3 Department of Intensive Care Medicine, The Affiliated Cancer Hospital of Zhengzhou University & Henan Cancer Hospital, Zhengzhou, China; 4 Department of Intensive Care Medicine, Henan Provincial People’s Hospital & Zhengzhou University People’s Hospital, Zhengzhou, China; 5 Department of Neurology, The Sixth Hospital of Wuhan, Affiliated Hospital of Jianghan University, Wuhan, China; 6 Department of Operation Room, Zhongnan Hospital of Wuhan University, Wuhan, China; 7 Department of Orthopedics, Union Hospital, Tongji Medical College, Huazhong University of Science and Technology, Wuhan, China; 8 Department of Intensive Care Medicine, Union Hospital, Tongji Medical College, Huazhong University of Science and Technology, Wuhan, China

**Keywords:** bibliometric analysis, diaphragmatic ultrasound, intensive care, machine learning, mechanical ventilation, rapid shallow breathing index, weaning

## Abstract

**Objective:**

To evaluate the research landscape and emerging trends in weaning from mechanical ventilation in ICU patients, providing insights to inform evidence-based practices in critical care nursing.

**Design:**

Bibliometric analysis.

**Methods:**

A systematic search identified 5,101 English-language studies published from January 2014 to February 2025 across the Web of Science Core Collection, PubMed, and Scopus. Data were analyzed using CiteSpace 6.4. R1 and VOSviewer to map publication trends, international collaborations, leading authors and institutions, core journals, and keyword clusters.

**Results:**

The volume of publications increased notably from 2016, peaking in 2021–2022. The United States, China, and France emerged as the top contributors, with key institutions such as the University of Toronto playing pivotal roles in collaborative networks. Research primarily focused on factors influencing weaning outcomes, evaluation of weaning criteria, and challenges associated with difficult weaning and extubation failure. Moreover, emerging trends emphasize the integration of diaphragmatic ultrasound, the rapid shallow breathing index (RSBI), and machine learning techniques to enhance predictive accuracy and optimize weaning strategies.

**Conclusion:**

The evolving research trends highlight a growing emphasis on multimodal assessment and advanced predictive tools in mechanical ventilation weaning. These findings offer valuable guidance for critical care nurses and multidisciplinary teams aiming to refine weaning protocols and improve patient outcomes in intensive care settings.

## Highlights

- First Bibliometric Analysis of ICU Ventilator Weaning Research (2014–2025): This decade-long analysis, employing CiteSpace and VOSviewer, identifies diaphragm ultrasound, rapid shallow breathing index (RSBI), and machine learning models as pivotal themes, underscoring their growing role in predicting extubation success and optimizing clinical decision-making.
- Global Collaboration Dominance Highlights Resource Disparities: The United States, China, and France lead global contributions, revealing stark geographic imbalances in research output and clinical implementation, necessitating equitable resource allocation and standardized interdisciplinary guidelines.
- Dynamic Multimodal Strategies to Mitigate Weaning Failure: Innovative approaches integrating real-time diaphragmatic thickening fraction, lung ultrasound, and systematic case report analyses show promise in refining protocols and addressing heterogeneity in critically ill populations to reduce failure rates.

## 1 Introduction

Mechanical ventilation (MV) is a fundamental life-support therapy widely used in intensive care units (ICUs) to support patients with severe respiratory failure or critical illness. Successful weaning from mechanical ventilation represents a crucial step in patient recovery. However, the weaning process remains complex and is influenced by multiple factors. Zhao et al. identified 19 variables associated with weaning failure, highlighting the multifactorial nature of ventilator liberation (1). In addition, regional differences in healthcare systems, technological support, and workforce structures may further influence weaning practices and outcomes (2). Previous studies have also shown that respiratory therapist–driven ventilation care bundles can improve adherence to lung-protective ventilation strategies in ICU settings (3).

Timely liberation from mechanical ventilation is essential because delayed or failed weaning is associated with adverse clinical outcomes. Among ICU patients eligible for weaning, the incidence of delayed weaning is approximately 9.6%, the failure rate reaches 15.6%, and mortality can reach 28.3% (4). Prolonged mechanical ventilation may also lead to significant patient discomfort and psychological distress, as dyspnea is often reported as one of the most distressing symptoms experienced by ventilated patients (5–6). Furthermore, failed weaning attempts may increase patients’ fear and anxiety, which can further impair respiratory function and negatively affect recovery outcomes (7).

Scientific knowledge mapping is a powerful visualization tool for revealing the structure, patterns, and distribution of scientific knowledge and its development (8). Bibliometric analysis is a quantitative research method that evaluates the development, structure, and trends of scientific literature through statistical analysis of publications, citations, and collaborative networks. In recent years, bibliometric approaches have been increasingly applied in medical and health sciences to identify research hotspots, emerging topics, influential authors, and global collaboration patterns. Compared with traditional narrative reviews, bibliometric analysis enables a more objective and systematic exploration of knowledge structures and research dynamics within a specific field.

Among the available bibliometric tools, CiteSpace and VOSviewer are two of the most widely used visualization software programs. CiteSpace focuses on detecting emerging trends and intellectual turning points through co-citation analysis, burst detection, and timeline visualization, while VOSviewer is particularly effective in constructing and visualizing large-scale bibliometric networks, including co-authorship, co-citation, and keyword co-occurrence relationships. These tools have been widely used in previous bibliometric studies across diverse medical disciplines, such as critical care, respiratory medicine, and nursing research. To ensure comprehensive coverage of the relevant literature, major bibliographic databases widely used in bibliometric research, including Web of Science Core Collection and Scopus, were considered in this study. These databases provide extensive citation indexing and have been widely used in previous bibliometric studies to explore scientific knowledge structures and research trends (9).

Therefore, the present study employed bibliometric methods to systematically analyze the global research landscape of mechanical ventilation weaning in ICU patients over the past decade. By utilizing CiteSpace and VOSviewer to construct knowledge maps, we aimed to identify publication trends, key contributors, research hotspots, and emerging directions in this field, thereby providing evidence-based insights for future research and clinical practice.

## 2 Methods

### 2.1 Data source

This study systematically retrieved relevant literature from the WOSCC, PubMed and Scopus databases, using a combination of controlled vocabulary and free-text terms to identify studies on weaning from mechanical ventilation in ICU patients. All document types indexed in the databases were included in order to capture the full scope of scientific outputs related to mechanical ventilation weaning. The search strategy was: (“ICU” OR “critical illness” OR “intensive care unit*”) AND (“artificial ventilation*” OR “mechanical ventilation” OR “mechanical ventilations” OR “respirator* ventilation*”) AND (“weaning” OR “remove” OR “extubation” OR “ventilator liberation”). The full database-specific search strategies are provided in Supplementary Table S1. The retrieval period spanned from January 1, 2014, to February 28, 2025. Inclusion criteria: (1) Literature focused on weaning from mechanical ventilation in ICU patients; (2) Complete bibliographic information in English; (3) All types of document categories. Exclusion criteria: (1) Duplicate publications; (2) Non-academic publications such as books, yearbooks, patents, technological reports, newspapers; (3) Advertisements, such as notices for papers or calls for submissions; (4) Editorial materials or supplementary data; (5) Study protocols or trial registration reports when identifiable.

### 2.2 Literature screening

**Figure 1:**
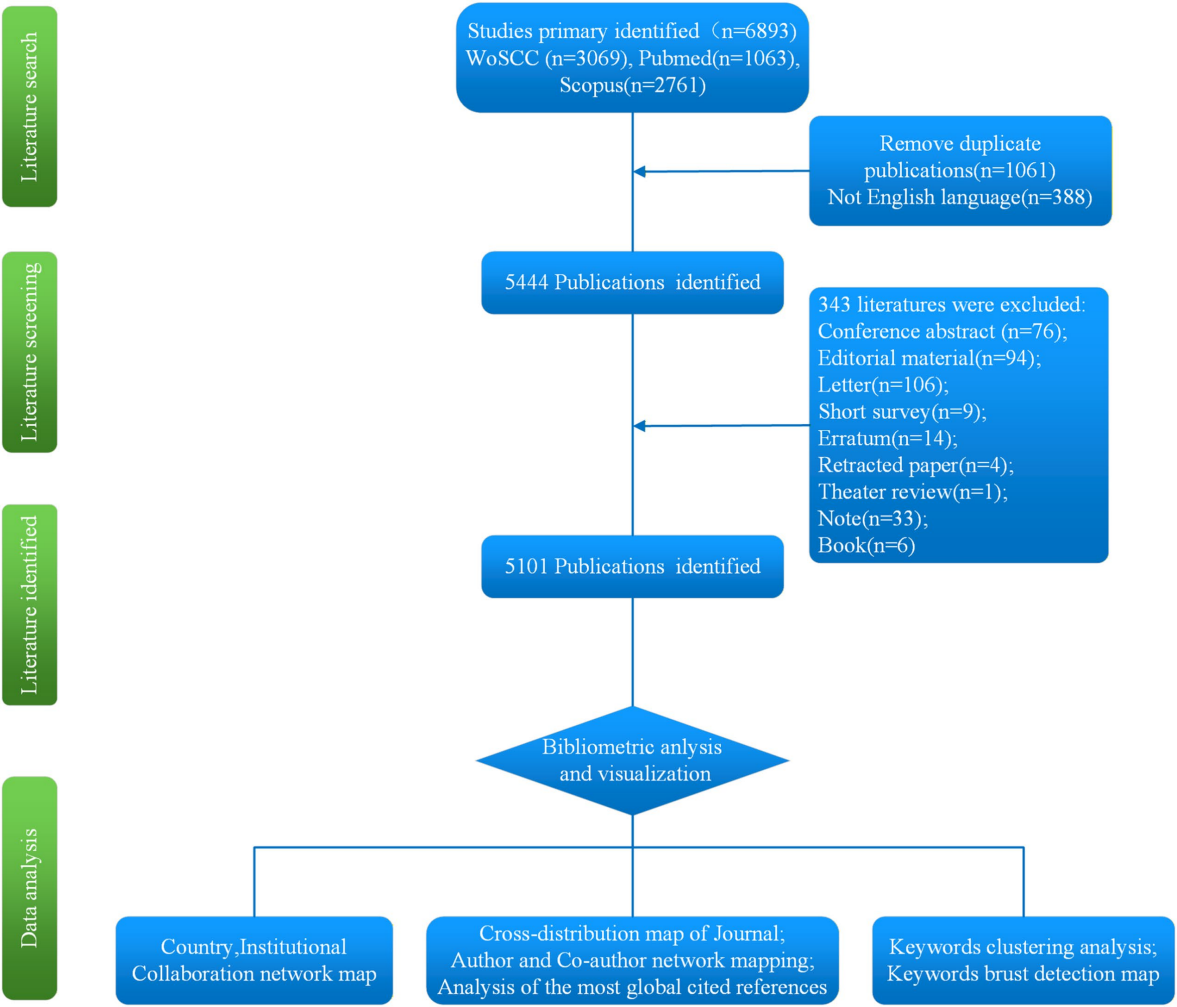
Flowchart of the study.

Two researchers independently conducted the literature search and screening. Disagreements were resolved through group discussions among the research team until consensus was reached. Retrieved articles were imported into NoteExpress reference management software for deduplication. Afterward, titles, keywords, and abstracts were manually screened to exclude irrelevant studies. For articles with conflicting inclusion decisions, full texts were reviewed, and the final decision was made collectively by the research team. The literature search, screening, identification and analysis method are illustrated in Figure 1. Bibliographic data of included studies for bibliometric analysis were exported in plain text format. The exported data included titles, authors, affiliations, abstracts, keywords, publication years, journals, volumes, and page numbers.

### 2.3 Data analysis

CiteSpace (version 6.4. R1) is a Java-based bibliometric visualization software developed by Chen for mapping knowledge structures and identifying emerging trends in scientific literature (10). It enables the construction of collaboration networks, co-citation networks, and keyword clusters, as well as the detection of citation bursts and timeline evolution of research topics. CiteSpace has been widely applied in bibliometric studies across multiple scientific disciplines to explore knowledge structures and research frontiers. In this study, CiteSpace was used to generate institutional collaboration networks, co-cited author networks, reference clustering maps, timeline visualizations, and keyword burst detection analyses. Co-citation networks were constructed based on the reference lists contained within the retrieved publications. CiteSpace identifies relationships among references that are cited together within the dataset, rather than relying on citation counts from individual databases. Therefore, differences in citation metrics across databases do not directly affect the co-citation network structure. VOSviewer, an R-based package, was employed for quantitative bibliometric and scientometric analysis, with an emphasis on data visualization (11). In this study, VOSviewer was used to generate national collaboration maps and global co-citation networks, offering intuitive visual representations to make academic findings more accessible and impactful.

### 2.4 Ethical approval

Because of the study was a retrospective review of previously published research, the Ethics committee approval was not required.

## 3 Results

### 3.1 Analysis of publication volume

**Figure 2:**
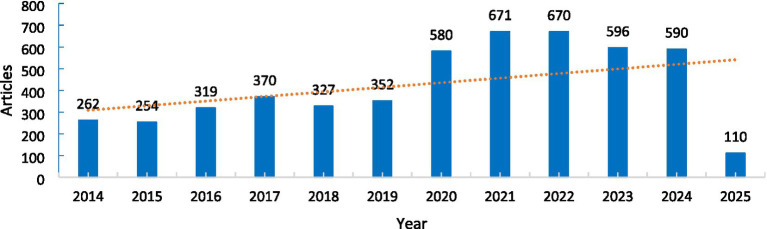
Trends in publications on ICU mechanical ventilation weaning.

A total of 5,101 English-language studies were included in this analysis. The eligible data within the specified timeframe were organized, and a publication trend chart was created, as shown in Figure 2. In 2016, the number of studies on “weaning from mechanical ventilation in ICU patients” has significantly increased. The highest publication volumes were observed in 2021 and 2022, with over 580 articles published each year. This indicates that the topic of ICU mechanical ventilation weaning has garnered significant attention from researchers in recent years.

### 3.2 Analysis of the global network of collaborations

**Figure 3:**
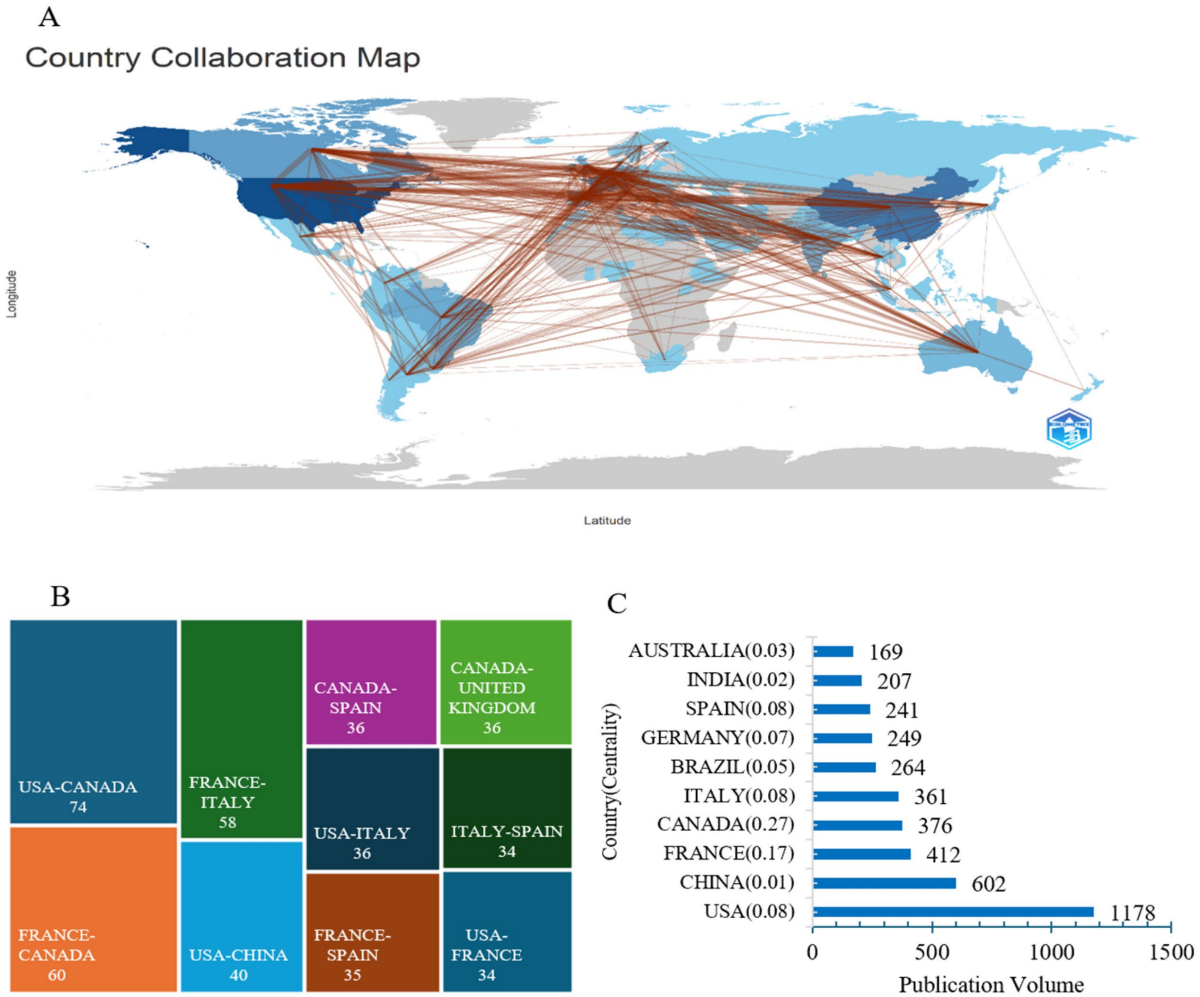
The global network of collaborations in ICU mechanical ventilation weaning research. **(A)** World Map of International Collaborations. **(B)** Countries’ collaborations and their frequency. **(C)** Publication volume and countries.

**Figure 4:**
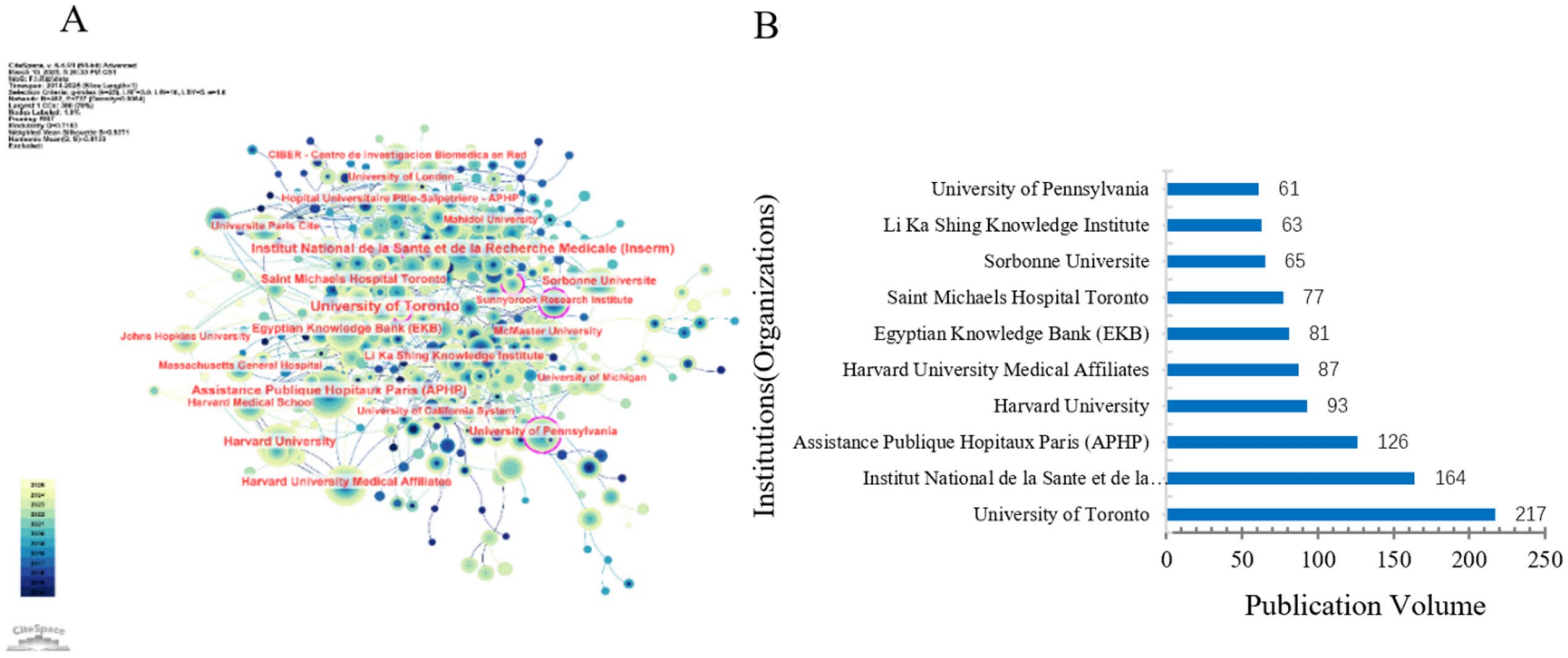
Analysis of global collaboration networks in research on ICU mechanical ventilation weaning. **(A)** Institutional collaboration network map. **(B)** The top 10 most productive institutions analysis.

The centrality index is a vital metric used in network analysis to assess the influence and importance of various entities—such as countries, institutions, authors—within an academic or research network (12). The centrality index, particularly betweenness centrality, is commonly used in bibliometric network analysis to measure the importance of a node within a collaboration or citation network. It reflects the extent to which a node lies on the shortest paths between other nodes in the network. A higher centrality value indicates that the node plays a bridging role in connecting different parts of the network. However, centrality values should be interpreted cautiously because they may be influenced by network size and data coverage. We illustrate the global network of collaborations in ICU mechanical ventilation weaning research. In Figure 3A, the darker the color, the greater the number of publications from a particular country or region. The frequency of connecting lines between countries indicates the intensity of collaboration between them. In Figure 3B, the most frequent collaborations occurred between the USA and Canada (74 instances), followed by France and Canada (60 instances), and France and Italy (58 instances). The top three countries with the highest publication volumes are the USA (1,178 publications), China (602 publications), and France (412 publications), as shown in Figure 3C. Overall, whether in terms of total collaboration strength or publication numbers, the USA significantly outpaces other countries, highlighting the importance of American authors in this field and their close collaborations with researchers from other countries. Figure 4A shows that the top three institutions with the highest publication volumes from abroad are *University of Toronto* (*n* = 217), *Institut National de la Santé et de la Recherche Médicale* (*n* = 164), and *Assistance Publique Hôpitaux Paris* (*n* = 126). Among the listed institutions, *University of Toronto* exhibits the highest centrality, indicating its significance as a key hub for collaborative connections, as shown in Figure 4B.

### 3.3 Journal analysis

**Figure 5:**
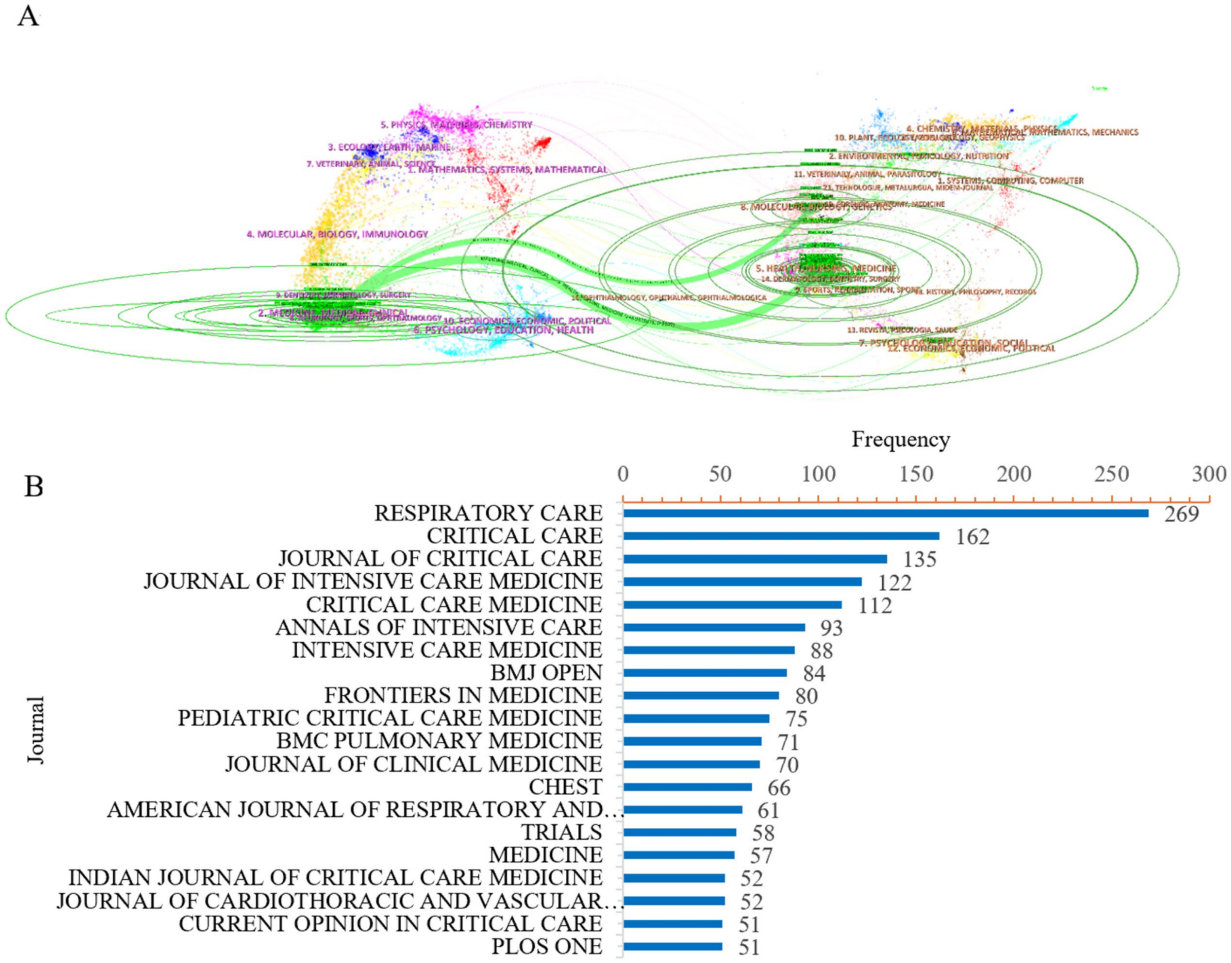
Analysis of journals in the field of ICU mechanical ventilation weaning. **(A)** Cross-disciplinary distribution map of journals based on the dual overlay method. **(B)** Core sources by Bradford’s law.

In the journal analysis, a dual overlay method was used to generate a cross-disciplinary distribution map of journals, which allows tracking the evolution of citation patterns and research hubs (13). In Figure 5A, the left side depicts the distribution of journals in the cited literature, while the right side shows the distribution of journals in the cited references, with paths connecting them through color coding. The analysis reveals that research on ICU mechanical ventilation weaning is primarily intersected with the fields of medicine, healthcare, and clinical research. These studies frequently cite literature from molecular biology, biology, genetics, health, and nursing. Using Bradford’s Law (14), a total of 20 journals were identified as publishing research on ICU mechanical ventilation weaning. In Figure 5B, the top three journals in terms of publication volume are *RESPIRATORY CARE* (*n* = 269), *CRITICAL CARE* (*n* = 162) and *JOURNAL OF CRITICAL CARE* (*n* = 135), reflecting the widespread interest in this topic among clinical readers and highlighting the significance of this research area.

### 3.4 Author and co-cited author analysis

**Figure 6:**
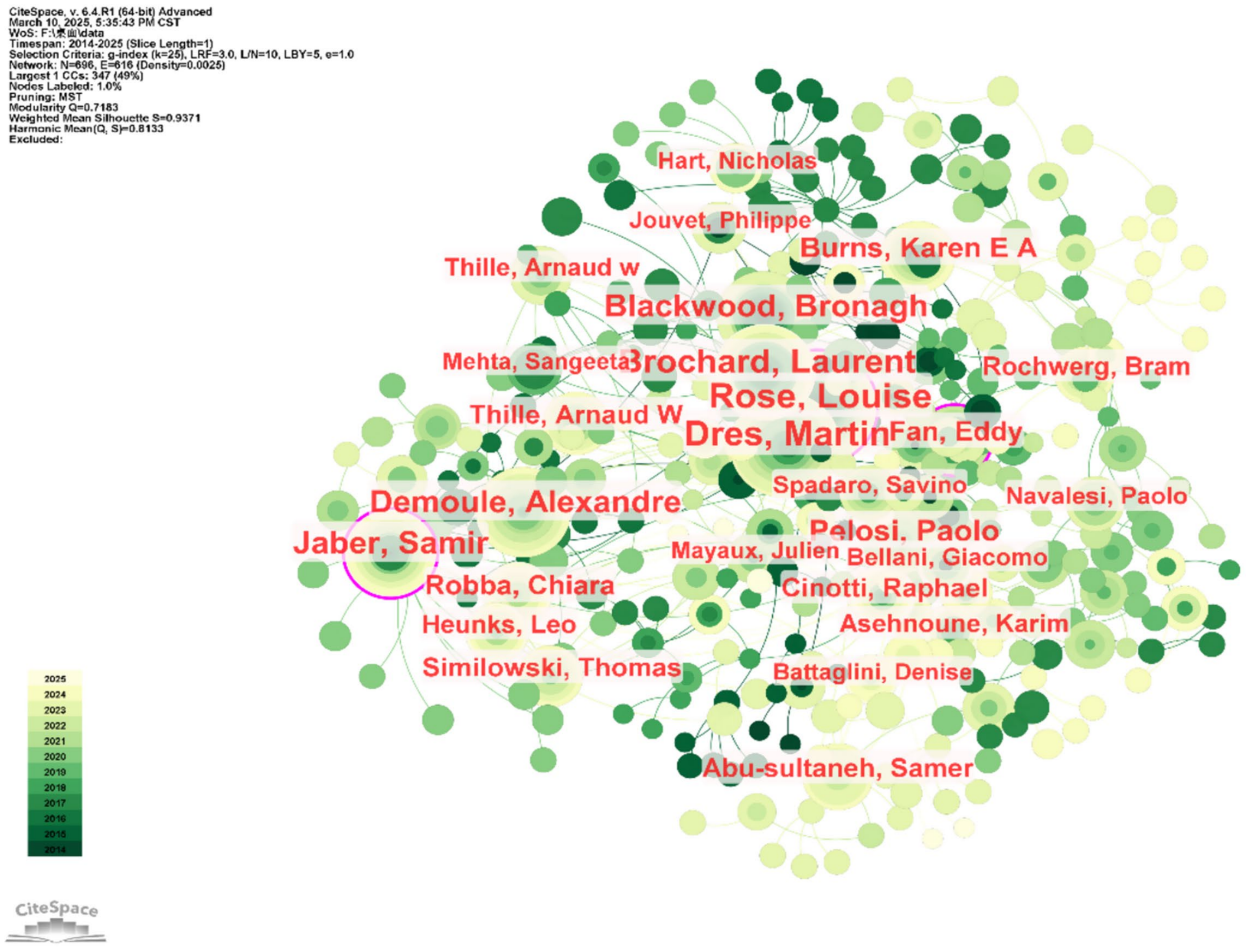
Author and co-author network mapping in ICU mechanical ventilation weaning research.

Cluster analysis was used to generate visualizations of the authors and co-cited authors in ICU mechanical ventilation weaning research. These visualizations aim to identify the most influential authors in this field. In Figure 6, each node represents an individual author. Table 1 lists the top 10 authors in descending order based on publication frequency and citation frequency. The analysis revealed that ROSE LOUISE (*n* = 64) is the most prolific author, with a centrality greater than 0.06. However, the highest-cited author is ESTEBAN A, with the highest Citation Frequency (*n* = 657). Both DRES MARTIN and JABER SAMIR appear among the top 10 in terms of publication volume and co-citation frequency. Notably, PELOSI PAOLO has a centrality score of 0.13, indicating a high level of influence and importance in this field.

**Table 1:** Top 10 authors and their citation frequencies in ICU mechanical ventilation weaning research.

Ranking	Authors	Frequency	Centrality	Cited author	Frequency	Centrality
1	ROSE LOUISE	64	0.07	ESTEBAN A	744	0.01
2	DRES MARTIN	60	0.06	BOLES JM	663	0.01
3	BROCHARD LAURENT	49	0.08	THILLE AW	615	0.02
4	DEMOULE ALEXANDRE	41	0.01	EPSTEIN SK	451	0.01
5	BLACKWOOD BRONAGH	41	0.03	ELY EW	431	0.02
6	JABER SAMIR	39	0.09	GOLIGHER EC	406	0.02
7	PELOSI PAOLO	31	0.13	DRES MARTIN	372	0.02
8	BURNS KAREN EA	25	0.06	GIRARD TD	356	0.02
9	FAN EDDY	23	0.09	MACINTYRE NR	353	0.01
10	ROBBA CHIARA	22	0.01	JABER SAMIR	344	0.02

### 3.5 Co-cited reference analysis

#### 3.5.1 Exploring Frontiers through globally co-cited reference

**Figure 7:**
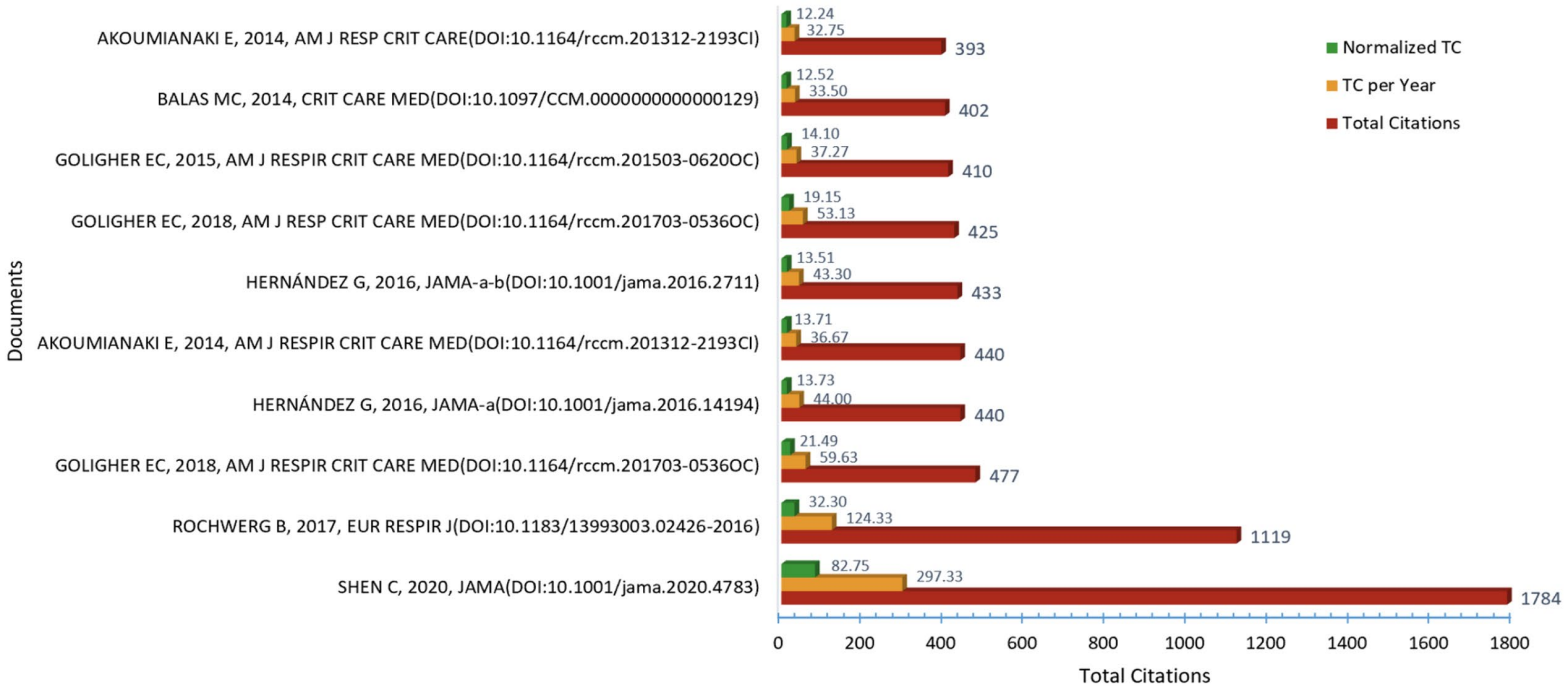
Analysis of most global cited references in ICU mechanical ventilation weaning.

We conduct a global co-citation reference analysis by using VOSviewer. The references are listed in descending order based on total citation count. In Figure 7, it includes the journal of publication, DOI, Total Citations (TC), Total Citations per Year (TC per Year), and Normalized Total Citations (Normalized TC). The three most highly cited papers are: SHEN C, 2020, JAMA (*n* = 1784), ROCHWERG B, 2017, EUR RESPIR J (*n* = 1,119), GOLIGHER EC, 2018, AM J RESPIR CRIT CARE MED (*n* = 477). Through an in-depth analysis of three references with over 450 citations, all of these studies have considered successful ventilator weaning as a clinically significant outcome, regardless of whether the focus was on ARDS patients or critically ill patients. Some highly cited references identified in the co-citation network were related to respiratory support strategies during the COVID-19 pandemic. Although these studies did not directly focus on ventilator weaning, they were frequently cited alongside ventilation-related research due to the substantial increase in critical care studies during the pandemic. SHEN C et al.’s study indicated that during treatment with antiviral drugs and methylprednisolone, the intravenous infusion of plasma from recovered COVID-19 patients can decrease viral loads and shorten the duration of mechanical ventilation in COVID patients with ARDS (15). Rochwerg B et al.’s clinical practice guideline provides European Respiratory Society/American Thoracic Society(ERS/ATS) recommendations for the clinical application of non-invasive mechanical ventilation(NIV). The guideline suggest that NIV should not be used in the treatment of patients with established post-extubation respiratory failure, but it can be used to facilitate weaning from mechanical ventilation in patients with hypercapnic respiratory failure (16). Goligher EC et al.’s study reported that the development of diaphragm atrophy during mechanical ventilation was significantly correlated with prolonged weaning periods and an elevated risk of severe complications, such as reintubation, tracheostomy, and extended ventilator dependence (17).

#### 3.5.2 Exploring hotspots through keywords clustering and timeline maps

**Figure 8:**
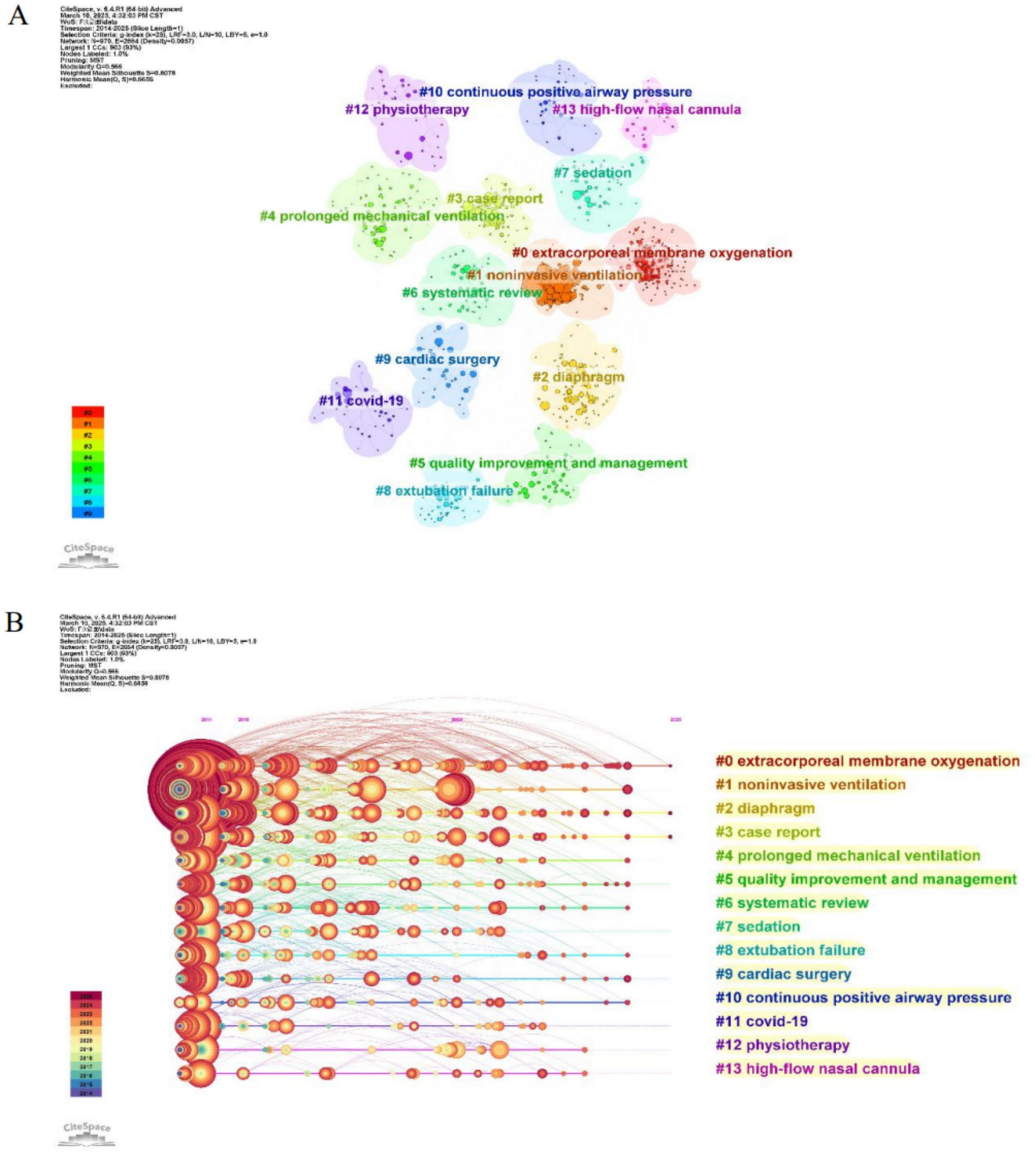
Analysis of keyword clusters in ICU research on mechanical ventilation weaning: **(A)** Keywords clustering map and **(B)** Keywords clustering timeline map.

Keywords cluster analysis is utilized to group similar or related keywords which facilitates the identification of research themes trends and potential areas for exploration. This method enables researchers to gain a comprehensive understanding of the organization of the literature and the evolution of various topics (18). In this study keywords cluster analysis was used to create keywords clustering and timeline maps yielding clusters with significant structure and high reliability (Q = 0.566 S = 0.808). In Figure 8A it displays the keywords clustering for ICU mechanical ventilation weaning research. Each cluster represents a specific research theme or area. By examining the different clusters and their associated keywords one can gain insights into the research hotspots within that field. In Figure 8B it shows the timeline map of keywords clustering where clusters are displayed along a time axis. This timeline map provides insight into the evolution of research themes and how different clusters have emerged and evolved over time. It highlights the active research areas such as “extracorporeal membrane oxygenation” “noninvasive ventilation” “diaphragm” “case report” “prolonged mechanical ventilation” “quality improve and management.”

#### 3.5.3 Exploring trends and directions through the keyword burst detection map

Burst detection is employed to identify phenomena characterized by abrupt surges or declines in variable values (e.g., citation counts or term frequencies) within short temporal intervals (17). This analytical method enables researchers to detect rapid shifts and emerging trends within scholarly domains, offering critical insights into dynamic changes in research focus or technological innovation. By using burst detection analysis, the keyword burst detection map for ICU mechanical ventilation weaning research was generated. The burst keywords that emerged over the past five years are primarily “shallow breathing index” “lung ultrasound” “case report” “machine learning,” as shown in Table 2. These keywords represent the main areas of research interest and future trends in the field.

**Table 2:** Detection of keyword bursts in ICU research on mechanical ventilation weaning.

Keywords	Year	Strength	Begin	End	2014–2025
positive end expiratory pressure	2014	44.88	2014	2019	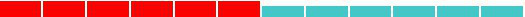
adverse effects	2014	23.16	2014	2017	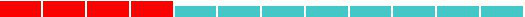
time factors	2014	17.98	2014	2019	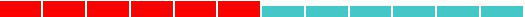
very elderly	2014	13.11	2014	2019	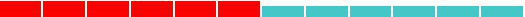
hypnotics and sedatives	2014	11.5	2014	2018	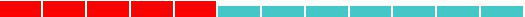
hypnotic sedative agent	2014	11.03	2014	2018	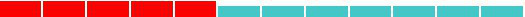
endotracheal tube	2014	10.89	2014	2017	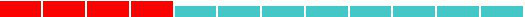
devices	2014	10.01	2014	2020	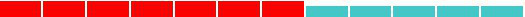
controlled trial	2014	9.72	2014	2017	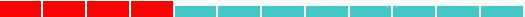
obstructive pulmonary disease	2014	8.86	2014	2018	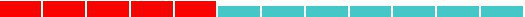
priority journal	2014	59.75	2015	2020	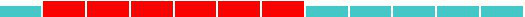
statistics and numerical data	2014	36.99	2015	2018	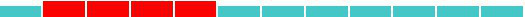
standards	2015	20.08	2015	2018	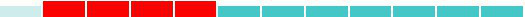
time factor	2015	16.65	2015	2019	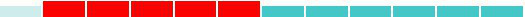
pathophysiology	2014	11.88	2015	2019	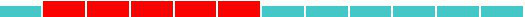
very elderly	2014	13.11	2014	2019	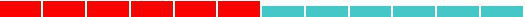
coronavirus disease 2019	2020	8.66	2020	2023	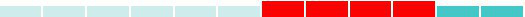
shallow breathing index	2020	27.63	2021	2025	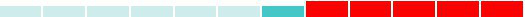
lung ultrasound	2020	25.62	2021	2025	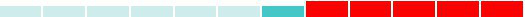
case report	2021	15.11	2021	2025	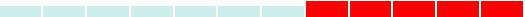
machine learning	2021	8.6	2021	2025	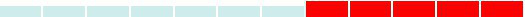

## 4 Discussion

### 4.1 Research status

Research on weaning from mechanical ventilation in ICU patients has received widespread attention from scholars. As shown in Figure 2, the number of studies on this topic has been steadily increasing. The United States, China, and France with the centrality score 0.08, 0.01and 0.17, have made significant contributions in terms of publication volume in the field of mechanical ventilation weaning. French research institutions have performed well in both publication volume and centrality, which may be attributed to France’s strong economy, healthcare system, and academic environment. Mechanical ventilation is one of the critical treatment methods for critically ill patients. However, as the duration of mechanical ventilation increases, the associated risks of complications also rise. Diaphragm dysfunction may lead to longer ICU stays, weaning failure, and increased mortality. Prolonged mechanical ventilation can further worsen diaphragm dysfunction, creating a vicious cycle where both factors interact, ultimately leading to ventilator dependence, extending mechanical ventilation time, and increasing hospitalization duration (19). These issues can negatively impact patient outcomes. The study by Leung C et al. (2) indicates that the applicability of weaning protocols for mechanically ventilated patients depends on social and cultural contexts. It is influenced by factors such as regional economic conditions, technological support, research capabilities, and the availability of respiratory therapists. These factors may contribute to the disparities observed between countries. Economic strength, high-quality healthcare resources, and prestigious academic institutions provide a solid talent pool for research in this field, presenting favorable opportunities for the development of mechanical ventilation weaning strategies. Therefore, it is recommended that national healthcare policies focus on supporting relevant scientific research and provide assistance to help balance the development of weaning research across countries. This would ensure the equitable and high-quality growth of the field.

### 4.2 Research hotspots

#### 4.2.1 Factors influencing mechanical ventilation weaning

The factors influencing mechanical ventilation weaning can be categorized into patient disease factors, healthcare provider cognitive factors, and mechanical ventilation parameter factors. A systematic review by Trudzinski et al. (20) identified several factors influencing weaning, including the patient’s comorbidities, intubation site, various laboratory test results, and ventilator settings. For patients on long-term mechanical ventilation, the impact on the respiratory system must also be considered. In Saeed F’s study (21), a stepwise weaning strategy was recommended for such patients, such as gradually reducing ventilator support or progressively increasing the duration of spontaneous breathing.

#### 4.2.2 Weaning indicator assessment

Thorough pre-weaning assessment is essential in effectively preventing the need for re-intubation. In 2017, the American Thoracic Society and the American College of Chest Physicians jointly updated and revised the *Weaning Guidelines for Critically Ill Patients* (22)based on the latest scientific evidence. These guidelines emphasize the importance of early assessment and planning for weaning, including evaluating the patient’s condition, setting appropriate weaning goals, selecting weaning methods, monitoring respiratory mechanics during the weaning process, assessing post-weaning outcomes, and ensuring clear communication with the patient and family to improve clinical weaning outcomes. Diaphragm function is a critical indicator in the weaning process. Mahmoodpoor A et al. (23) conducted a systematic review that highlighted diaphragm thickness rate assessment as superior to diaphragm displacement. The reason for this is that diaphragm thickness rate reflects the active contraction state of the diaphragm during mechanical ventilation, whereas diaphragm displacement is correlated with tidal volume. The sensitivity and specificity of diaphragm thickness rate are higher than those of diaphragm displacement. Given this, for patients requiring weaning, operators should not only focus on the patient’s overall condition, ventilator settings, and respiratory mechanics monitoring, but also consider factors that influence these parameters, particularly diaphragm function. Specifically, diaphragm thickness rate may serve as a more valuable predictor of successful weaning.

#### 4.2.3 Difficult weaning and weaning failure

Globally, the causes of mechanical ventilation weaning failure involve complex multisystem interactions. Common risk factors include respiratory system impairments (24) (e.g., diaphragmatic atrophy, increased airway resistance in COPD patients), cardiovascular complications (25) (with weaning-induced pulmonary edema accounting for 59% of SBT failures), and neurological dysfunction (26) (patients with GCS < 8 have a 38% higher extubation failure rate than those with GCS ≥ 8). Psychological conditions such as depression, anxiety, or delirium can hinder successful weaning and increase the risk of extubation failure. Patients may develop these psychological issues due to factors such as unfamiliarity with the medical environment and fear of their illness, further contributing to weaning difficulties. A study by Jubran A et al. (27) demonstrated that patients with depression have a higher risk of weaning failure. Regional variations are significant. A study conducted in the medical intensive care unit of a 325-bed teaching hospital in the United States found that extubation failure rate reached 100% in patients with the following risk factors: reduced peak expiratory flow (PEF ≤ 60 L/min), increased sputum production (>2.5 mL/h), and neurological impairment (inability to follow commands) (26). In contrast, patients without any of these risk factors had an extubation failure rate of only 3%. In China, the overall SBT failure rate is 45%, with 59% attributed to pulmonary edema (25). In Portugal, standardized weaning protocols have reduced reintubation rates from 48 to 17% (28), whereas in developing countries, limited resources (e.g., inadequate infection control and nutritional imbalances) increase reintubation risk by 10–15% (29). In specific populations, neuromuscular disease patients (e.g., Guillain-Barré syndrome) had an extubation failure rate of 22.73% (30), which was associated with autonomic dysfunction in the ICU and diaphragmatic atrophy due to prolonged bed rest. Future research should focus on enhancing mechanical ventilation weaning assessment strategies in different populations.

Spontaneous breathing trial (SBT) is widely used as an early weaning assessment tool for mechanically ventilated patients in clinical practice. However, SBT depends on clinical signs and physiological parameters, which can be influenced by factors such as sedation, diaphragm fatigue, and psychological factors. A key challenge in current research is how to optimize the parameters and indicators of the SBT, combining them with other assessment tools to improve the accuracy of weaning predictions for ICU mechanical ventilation patients. Ventilator-induced diaphragmatic dysfunction (VIDD) is one of the major causes of difficult weaning in mechanically ventilated patients (31). When weaning failure is determined to be caused by respiratory muscle dysfunction, medical teams should provide targeted, intensified treatment to improve outcomes (32). Given the complex nature of ICU mechanical ventilation patients’ conditions, it is recommended to include new indicators, such as neuromuscular function, peak expiratory flow rate, and laboratory parameters, into the weaning assessment. Additionally, employing a multi-parameter approach and multimodal imaging results can enhance the accuracy of weaning predictions, thereby increasing the success rate of weaning and reducing weaning-related mortality in ICU mechanical ventilation patients.

### 4.3 Future development trends

International scholars have paid significant attention to predictive indicators, weaning assessment methods, and research approaches related to mechanical ventilation weaning. Key research frontiers in the field of mechanical ventilation weaning include “shallow breathing index” “lung ultrasound” (diaphragm thickness assessment), “case report” and “machine learning.” The Rapid Shallow Breathing Index (RSBI) is one of the core indicators for pre-weaning assessment and is widely used to screen patients suitable for a spontaneous breathing trial (SBT). According to the 2016 Official American Thoracic Society/American College of Chest Physicians(ATS/ACCP) Clinical Practice Guideline (33) and the Indian Society of Critical Care Medicine(ISCCM) guideline (34), an RSBI of less than 105 breaths/min/L suggests a higher likelihood of successful extubation and is recommended as a fundamental parameter for weaning assessment. However, multiple studies have indicated significant variability in the sensitivity and specificity of RSBI, particularly in patients with chronic pulmonary disease or respiratory muscle fatigue, where its predictive value diminishes (35–37). This limitation may be attributed to RSBI being merely a respiratory parameter that does not reflect the overall health status of the patient. Therefore, it should ideally be used in conjunction with other indicators. Recent research has shown that bedside ultrasound assessment of diaphragmatic excursion (DE) and diaphragmatic thickening fraction (DTF) can predict extubation outcomes (38–40). Ultrasound plays an indispensable role in assessing diaphragm function status (41). Cappellini I et al. (42) focused on dynamically assessing weaning outcomes by monitoring diaphragm thickness rate, diaphragm displacement, or the diaphragm displacement time index. A study by Parada-Gereda et al. (43) demonstrated that patients with DE greater than 1 cm and DTF exceeding 29% had a higher success rate in weaning. Thus, incorporating diaphragmatic ultrasound as a supplementary tool in weaning assessments can reduce reliance on the RSBI alone, which may lower the failure rate of extubation in mechanically ventilated patients. This approach is closely related to the complexity of weaning assessments in the ICU and the variability in the clinical conditions of critically ill patients.

It highlights the necessity for ICU physicians to implement multimodal assessments, dynamic monitoring, and systematic management to optimize extubation strategies. ICU physicians should consider timely extubation once the patient’s underlying condition is stabilized. Before extubation, a comprehensive evaluation of vital signs, laboratory data, imaging, and respiratory parameters is essential. During the SBT, real-time bedside ultrasound monitoring of DE changes should be employed; if there is an increase in D-RSBI or if DE remains below the critical threshold, the risk of extubation failure must be cautiously evaluated. It is also important to closely monitor the patient’s ability to cough and consciousness level within the first 48 h post-extubation to promptly identify factors contributing to post-extubation hypoxemia and to minimize the likelihood of re-intubation. Additionally, regularly summarizing difficult or failed weaning cases in the form of case reports, along with analyzing the causes, can deepen healthcare providers’ understanding of mechanical ventilation weaning and help accumulate valuable weaning experience.

In recent years, case reports and machine learning–based studies have frequently appeared in the literature on mechanical ventilation weaning. While case reports may contribute valuable clinical observations, they generally represent a lower level of evidence and should be interpreted cautiously when informing clinical practice. In contrast, machine learning approaches are increasingly used as analytical tools to develop predictive models for extubation outcomes. These approaches can integrate large volumes of clinical data and may complement traditional statistical models in identifying predictors of weaning success. Machine learning (ML) methods have been widely applied in the research of mechanical ventilation weaning. Data related to weaning, such as patient demographics, laboratory and imaging indicators, and both intrinsic and extrinsic factors, are first collected and organized. The appropriate algorithm is then selected based on the data type, followed by model training and evaluation of the model’s effectiveness. Ultimately, scientifically validated models are applied clinically to improve the safety and effectiveness of mechanical ventilation weaning. Zhao QY et al. (1) used recursive feature elimination algorithms to select 19 key features from a database of 16,189 patients (including 2,756 failed extubations), which included age, BMI, stroke history, heart rate, respiratory rate, mean arterial pressure, peripheral oxygen saturation, temperature, pH value, central venous pressure, tidal volume, positive end-expiratory pressure (PEEP), mean airway pressure, pressure support ventilation level, mechanical ventilation duration, spontaneous breathing trial success count, urine output, crystalloid fluid volume, and antibiotic types. This provided valuable reference for clinical decision-makers to better understand patient conditions and develop appropriate treatment strategies. A systematic review by Huang K et al. (44) found that mechanical ventilation weaning management using machine learning algorithms could reduce ventilator-induced lung injury caused by conventional weaning methods and is particularly beneficial for patients with difficult weaning.

Successful liberation from mechanical ventilation remains a key challenge in critical care medicine, and improving the prediction of weaning outcomes continues to be a major research focus. In recent years, there has been a growing shift from single-parameter assessment toward multimodal monitoring approaches, which integrate respiratory mechanics, hemodynamic indicators, imaging findings, and clinical parameters to provide a more comprehensive evaluation of patients’ readiness for extubation.

Moreover, artificial intelligence (AI) and machine learning (ML) techniques are increasingly being applied to analyze complex physiological datasets and develop predictive models for ventilator weaning outcomes. Several studies have explored reinforcement learning–based models to optimize ventilator management strategies and support clinical decision-making. Feng et al. (45) developed an optimal regime model for making treatment decisions of discontinuing ventilation, continuing NIV, or intubation, and the model was applicable across various disease groups with distinguished achievement in dealing with respiratory disorders and improving treatment outcomes. In addition, dynamic time-series prediction models integrating physiological monitoring data have been developed to identify patients at risk of weaning failure. Acevedo HG et al. (46) applied Time-frequency analysis techniques to process time-series data from electrocardiogram and respiratory flow signals. Machine learning and convolutional neural network (CNN) classifiers were subsequently developed to assess patient readiness for successful extubation, achieving a model accuracy of 90.1%. These data-driven approaches have the potential to support more dynamic and individualized decision-making processes, ultimately contributing to the development of semi-automated or closed-loop weaning strategies.

## 5 Limitations

As the first bibliometric analysis of the ICU mechanical ventilation weaning topic, this study provides a comprehensive exploration of the relevant knowledge, offering a foundation for the scientific basis and future research directions in this field. We primarily utilized two mainstream bibliometric tools and leveraged their strengths for analysis. However, there are certain limitations to this study. First, our inclusion criteria only selected articles published in English, which may have overlooked non-English publications in the field. Second, this study included multiple document types, including original research articles and review articles. Review papers may receive higher citation counts and often involve collaborations among established experts from different institutions and countries. Therefore, their inclusion may have influenced patterns observed in institutional prominence and international collaboration networks. Third, the result of citation counts and co-citation networks do not necessarily reflect the quality or clinical impact of individual studies; rather, they merely indicate their visibility within the academic community. When using the results from these highly cited studies, it is essential to integrate clinical expertise and thoroughly consider the authenticity and reliability of the findings. Additionally, the focus of this study was specifically on the issues related to weaning from mechanical ventilation in critically ill patients, which may have neglected differences in types of mechanical ventilation. Another factor that may have influenced the publication trends observed in this study is the COVID-19 pandemic. The global surge in critically ill patients requiring mechanical ventilation during 2020–2021 stimulated an unprecedented increase in critical care research output. As a result, the growth in publications related to mechanical ventilation and weaning during this period may partly reflect this exceptional global event rather than a purely linear development of the research field. These differences could also be an important factor contributing to weaning failure. Therefore, future researchers may consider addressing these gaps and refining the analysis to account for such variations.

## 6 Conclusion

This study conducted a bibliometric analysis of ICU mechanical ventilation weaning literature using CiteSpace 6.4. R1 and VOSviewer software, providing targeted recommendations for future research and clinical practice. In terms of the current research status, the number of publications related to ICU mechanical ventilation weaning is showing a positive upward trend. From the analysis of high-frequency keywords and keyword clustering results, it is clear that ultrasound and mechanical ventilation are key research hotspots in this field. The main research focuses include factors influencing difficult weaning and weaning failure, ultrasound assessment, weaning process management in long-term mechanical ventilation patients, weaning success rates, and weaning-related mortality. Scholars have paid significant attention to predictive indicators for mechanical ventilation weaning, assessment methods, and research approaches.

Future researchers can further explore the application of machine learning methods in this field, focusing on mechanical ventilation weaning indicators, such as the rapid shallow breathing index and diaphragm thickness, to enhance the understanding and development of weaning strategies. This could help optimize the weaning process and improve the success rate of mechanical ventilation weaning.

## Data Availability

The original contributions presented in the study are included in the article/Supplementary material, further inquiries can be directed to the corresponding authors.
