# Supplementary material for: Weaning from mechanical ventilation in ICU patients: research hotspots and trends in the past decade—a bibliometric analysis
Source: Front Med (Lausanne). 2026 Apr 10;13:1790796. doi: 10.3389/fmed.2026.1790796 (PMC13105878; doi:10.3389/fmed.2026.1790796)
Supplement: Supplementary file 1 [file Table_1.docx]

**Literature Search Strategy**

**(1) Web of Science**

| **Search number** | **Search Query** | **Results** |
| --- | --- | --- |
| 1 | intensive care unit* (Topic) OR critical care (Topic) OR critical ill* (Topic) OR ICU (Topic) Timespan: 2014-01-01 to 2025-02-28 | 307397 |
| 2 | weaning* (Topic) OR liberation (Topic) OR extubat* (Topic) OR difficult* weaning* (Topic) OR weaning failure (All Fields) OR weaning assessment (All Fields) OR weaning management (All Fields) Timespan: 2014-01-01 to 2025-02-28 | 49833 |
| 3 | Artificial Respiration* (Topic) OR Mechanical Ventilation* (Topic) OR Respitator* ventilation* (Topic) Timespan: 2014-01-01 to 2025-02-28 | 48465 |
| 4 | #1 AND #2 AND #3 | 3069 |

**(2) PubMed**

| **Search number** | **Query** | **Results** |
| --- | --- | --- |
| #1 | "Intensive Care Units"[Mesh] | 115,009 |
| #2 | ((critical* ill*[Title/Abstract]) OR (critical ill patient*[Title/Abstract])) OR (ICU[Title/Abstract]) | 154,441 |
| #3 | "Ventilator Weaning"[Mesh] | 4,754 |
| #4 | (((((Weaning, Ventilator[Title/Abstract]) OR ( ventilator liberation[Title/Abstract])) OR (Mechanical Ventilator Weaning[Title/Abstract])) OR (Ventilator Weaning, Mechanical[Title/Abstract])) OR (Respirator Weaning[Title/Abstract])) OR (Weaning, Respirator[Title/Abstract]) | 294 |
| #5 | (((weaning failure[Title/Abstract]) OR (difficult weaning[Title/Abstract])) OR (weaning assessment[Title/Abstract])) OR (weaning management[Title/Abstract]) | 938 |
| #6 | "Respiration, Artificial"[Mesh] | 93,115 |
| #7 | ((((((Artificial Respiration[Title/Abstract]) OR (Artificial Respirations[Title/Abstract])) OR (Respirations, Artificial[Title/Abstract])) OR (Ventilation, Mechanical[Title/Abstract])) OR (Mechanical Ventilations[Title/Abstract])) OR (Ventilations, Mechanical[Title/Abstract])) OR (Mechanical Ventilation[Title/Abstract]) | 72,288 |
| #8 | #1 OR #2 | 220,983 |
| #9 | #3 OR #4 OR #5 | 5,397 |
| #10 | #6 OR #7 | 133,564 |
| #11 | #8 AND #9 AND #10 | 2,007 |
| #12 | "(2014/1/1-2015/2/28[publication date])" | 1,063 |

**（3）Scopus**

| Search number | Query | Results |
| --- | --- | --- |
| 1 | ( ( TITLE-ABS-KEY ( ICU ) OR TITLE-ABS-KEY ( intensive AND care AND unit ) OR TITLE-ABS-KEY ( critical AND illness ) OR TITLE-ABS-KEY ( critical AND care ) OR TITLE-ABS-KEY ( critically AND ill AND patient ) ) ) | 650,498 |
| 2 | ( ( TITLE-ABS-KEY ( weaning ) OR TITLE-ABS-KEY ( ventilator AND liberation ) OR TITLE-ABS-KEY ( ventilator AND weaning ) OR TITLE-ABS-KEY ( weaning AND from AND mechanical AND ventilation ) OR TITLE-ABS-KEY ( mechanical AND ventilator AND weaning ) OR TITLE-ABS-KEY ( respirator AND weaning ) OR TITLE-ABS-KEY ( weaning AND failure ) OR TITLE-ABS-KEY ( difficult AND weaning ) OR TITLE-ABS-KEY ( weaning AND assessment ) OR TITLE-ABS-KEY ( weaning AND management ) ) ) | 70,874 |
|  | ( ( TITLE-ABS-KEY ( mechanical AND ventilation ) OR TITLE-ABS-KEY ( mechanical AND ventilations ) OR TITLE-ABS-KEY ( artificial AND respiration ) OR TITLE-ABS-KEY ( artificial AND respirations ) ) ) | 134,838 |
| 3 | #1 AND #2 AND #3 | 5,058 |
| 4 | PUBYEAR > 2014 AND PUBYEAR < 2025 | 3,021 |
| 5 | ( LIMIT-TO ( SUBJAREA , "MEDI" ) OR LIMIT-TO ( SUBJAREA , "NURS" ) | 2,862 |
| 6 | ( LIMIT-TO ( SRCTYPE , "j" ) ) | 2446 |
